# Development and validation of a predictive nomogram for response to biologics and targeted therapy in Crohn’s disease: a retrospective cohort study

**DOI:** 10.3389/fimmu.2026.1788290

**Published:** 2026-04-16

**Authors:** Ping Lin, Wei Wang, Yun Zhou, Yong Yang, Ping Liu

**Affiliations:** The Department of Gastroenterology, The First People’s Hospital of WuHu, Wuhu, Anhui, China

**Keywords:** Clinical remission, Crohn’s disease, nomogram, predictive factors, upadacitinib

## Abstract

**Background:**

Biologics and targeted therapies are a first-line treatment option for individuals with Crohn’s disease (CD). Nevertheless, clinical remission rates vary considerably among different biologics and targeted therapies. This study aims to develop and validate a predictive nomogram for the response to biologics and targeted therapy in individuals with CD.

**Methods:**

This study included individuals with CD admitted to the First People’s Hospital of Wuhu from January 2020 to June 2025. Clinical remission rates at 14 and 26 weeks were observed. The clinical remission rates of different biologics and targeted therapy were analyzed using the chi-square test, and multiple comparisons were performed. The least absolute contraction and selection operator (LASSO) regression algorithm was used to determine variables related to clinical remission. A nomogram model was constructed, and the model performance was assessed utilizing receiver operating characteristic (ROC) curves, calibration curves, and decision curves (DCA). Internal validation was performed using repeated sampling and Harrell’s concordance index (C-index).

**Results:**

218 patients were included in the study. Compared with other biologics, upadacitinib (UPA) had the highest clinical remission rate at week 26 (94.1%,16/17subjects). Nine variables were identified by LASSO regression, including pre-treatment Crohn’s disease activity index (CDAI), disease duration, smoking history, disease site, disease behavior, perianal lesions, hemoglobin (Hb), erythrocyte sedimentation rate (ESR), and type of biologic. The area under the curve (AUC value) was 0.796. The calibration curve and DCA curve showed good calibration and clinical applicability. The internal validation also demonstrated moderate discriminative performance, with a C-index of 0.74.

**Conclusions:**

The study results demonstrate that UPA appears to show a significant clinical remission rate in a small cohort, but this finding needs to be validated in a larger-scale study. Furthermore, a practical tool has been developed to help clinicians identify the treatment response to biologics and targeted therapy in CD patients, promoting the development of personalized treatment and precision medicine.

## 1 Introduction

Crohn’s disease (CD) represents a chronic, recurrent disease that may involve the entire digestive tract, with unknown etiology. Its main clinical manifestations encompass abdominal pain, diarrhea, and weight loss, often accompanied by systemic symptoms like fever and fatigue, local manifestations like perianal abscesses or anal fistulas (1), and extraintestinal damage to joints, skin, eyes, and oral mucosa. It can also lead to serious complications such as intestinal obstruction, fistulas, and abdominal abscesses (2). These complications reduce patients’ quality of life, cause physical and psychological illnesses, and increase the risk of mortality (3, 4). Recurrent symptoms and frequent hospitalizations due to CD place a heavy medical and economic burden on society and families.

CD is primarily treated with drug therapy. Nonetheless, the efficacy of traditional drugs such as aminosalicylic acids, glucocorticoids, and immunosuppressants is not ideal. With in-depth research into the pathogenesis of CD, biologics and small-molecule targeted drugs have emerged for the clinical treatment of CD. Drugs such as infliximab (IFX), ustekinumab (UST), vedolizumab (VDZ), and upadacitinib (UPA) offer more options for individualized treatment of CD patients. However, their efficacy still varies significantly among different individuals (5). Furthermore, since these new drugs have been used only for a short time in clinical settings, it is imperative to explore their long-term efficacy and safety. Given the differences in clinical symptoms, disease severity, and genetic factors, the treatment response to drugs in CD patients varies. Comparing the efficacy of different drugs is necessary to understand their applicability in different patient populations. Meanwhile, drugs with the same efficacy but lower cost can help alleviate the economic burden on patients and coordinate the use of health resources.

Existing research primarily focuses on comparing the efficacy of IFX with UST or VDZ (6–8), while the number of real-world studies related to UPA remains relatively limited. Therefore, this study aims to compare the efficacy of four therapeutic regimens, including IFX, UPA, UST, and VDZ, and to construct and internally validate a nomogram model for predicting the clinical remission in CD patients. The goal is to assist clinicians in developing personalized treatment strategies, optimizing treatment pathways, and reducing ineffective treatments and waste of medical resources.

## 2 Materials and methods

### 2.1 Study design and patients

This study is a retrospective observational cohort study. This study included inpatients diagnosed with CD at the Inflammatory Bowel Disease Center of First People’s Hospital of Wuhu from January 2020 to June 2025. The diagnosis of CD was determined according to internationally recognized standards, based on clinical manifestations, endoscopic or surgical examinations, radiological, histological, and serological results (9, 10).

#### 2.1.1 Inclusion criteria

(1) Individuals clinically diagnosed with CD; (2) Age ≥ 18 years. (3) Individuals receiving biologics or targeted therapy for more than 26 weeks with complete follow-up data. (4) Pre-treatment Crohn’s disease activity index (CDAI) ≥ 150 points, and pre-treatment Harvey-Bradshaw Index (HBI) > 4 points.

#### 2.1.2 Exclusion criteria

(1) Individuals with confirmed or suspected other gastrointestinal malignancies. (2) Individuals with concurrent severe organ dysfunction. (3) Individuals with incomplete clinical data. (4) Refusal or self-discontinuation of treatment.

#### 2.1.3 Ethical approval

This study was approved by the Clinical Research Ethics Committee of First People’s Hospital of Wuhu (No.: YYLL20250037) and was performed according to the principles of the Declaration of Helsinki. Informed consent was obtained from all participants.

### 2.2 Data collection

The following demographic data and clinical characteristics were collected from the patient’s electronic medical record system, mainly including the following four aspects. (1) Patient’s baseline information encompassed sex, age, course of disease, smoking history, previous use of glucocorticoids, body mass index (BMI), disease-related variables (age at diagnosis, lesion site, disease behavior, whether there is an anal fistula). (2) Laboratory test indicators included serum albumin (ALB), hemoglobin (Hb), platelet (PLT), C-reactive protein (CRP), erythrocyte sedimentation rate (ESR), plateletcrit (PCT), lymphocyte count (LYM), and platelet-to-lymphocyte ratio (PLR). (3) Treatment regimens included IFX, UST, VDZ, and UPA. (4) We also extracted CDAI scores before and after treatment, and Harvey-Bradshaw Index (HBI) before and after treatment. Both CDAI and HBI scores were assessed by two or more gastroenterologists according to hospitalization and follow-up records.

### 2.3 Endpoints

The endpoint was clinical remission following induction therapy. CDAI and HBI were used as the primary indicators for evaluating clinical remission (11). CDAI activity was defined as ≥150 points, and clinical remission was defined as CDAI <150 points. HBI activity was defined as an HBI score > 4 points, and clinical remission was defined as an HBI score ≤ 4 points. We chose these endpoints because many patients lacked baseline endoscopic data and fecal calprotectin data. Although CDAI and HBI are impacted by patient subjectivity, considering the realities in China, clinical remission is not only a preliminary treatment goal but also a crucial factor in ensuring patient adherence. Clinical remission was assessed at weeks 14 and 26 (12–15).

### 2.4 Study methods

#### 2.4.1 Statistical analysis

The Shapiro-Wilk test was adopted to test the normality of continuous variables. Normally distributed variables were expressed as mean ± standard deviation, while abnormally distributed variables were expressed as median (interquartile range). Categorical variables were expressed as frequency (percentage). Pearson’s chi-square test, Fisher’s exact test, or Kruskal-Wallis test was performed to examine baseline characteristics between different treatment groups. The chi-square test and multiple comparisons were used to ascertain the clinical remission rates of different therapeutic regimens. The repeated measures ANOVA was performed to investigate changes in disease activity before and after treatment.

#### 2.4.2 Analysis of prognostic factors

LASSO regression was used for variable selection. LASSO regression is a compression estimation technique based on L1 regularization. By introducing a penalty term to construct the objective function, it can avoid overfitting and solve serious collinearity problems. It is robust and a commonly used method for variable selection. In this study, the optimal penalty parameter (lambda, λ) was determined by 10-fold cross-validation. Two commonly used λ selection criteria were considered in the cross-validation: the logarithm of lambda corresponding to the minimum error (lambda.min), and the logarithm of lambda corresponding to the minimum deviation by one standard error (lambda.lse). Based on the principle of minimizing prediction error, this study extracted the variable corresponding to lambda.min.

#### 2.4.3 Construction and evaluation of nomogram

Multiple logistic regression analysis was performed on the selected variables in the LASSO regression model to build a predictive model. Then a nomogram was plotted to visualize the model. In the nomogram, a score was given to each independent variable as per the partial regression coefficient in the multivariate model. Then, the scores of each independent variable were summed to compute a total score. The predictive performance of individual outcome events was assessed based on the total score. This tool predicts the probability of outcome events in an intuitive and quantitative way (16). The discriminative performance of the model was assessed by calculating the area under the ROC curve (AUC) and Harrell’s concordance index (C-index), and calibration was examined by plotting a calibration curve. The clinical application value of the model was appraised by the DCA curve (17). The robustness of the model was internally verified using the bootstrap method (18).

#### 2.4.4 Statistical software

All data analyses were performed using R version 4.5.2 and SPSS version 27.0. A p-value < 0.05 indicated a statistically significant difference.

## 3 Results

### 3.1 Baseline information of patients

**Figure 1:**
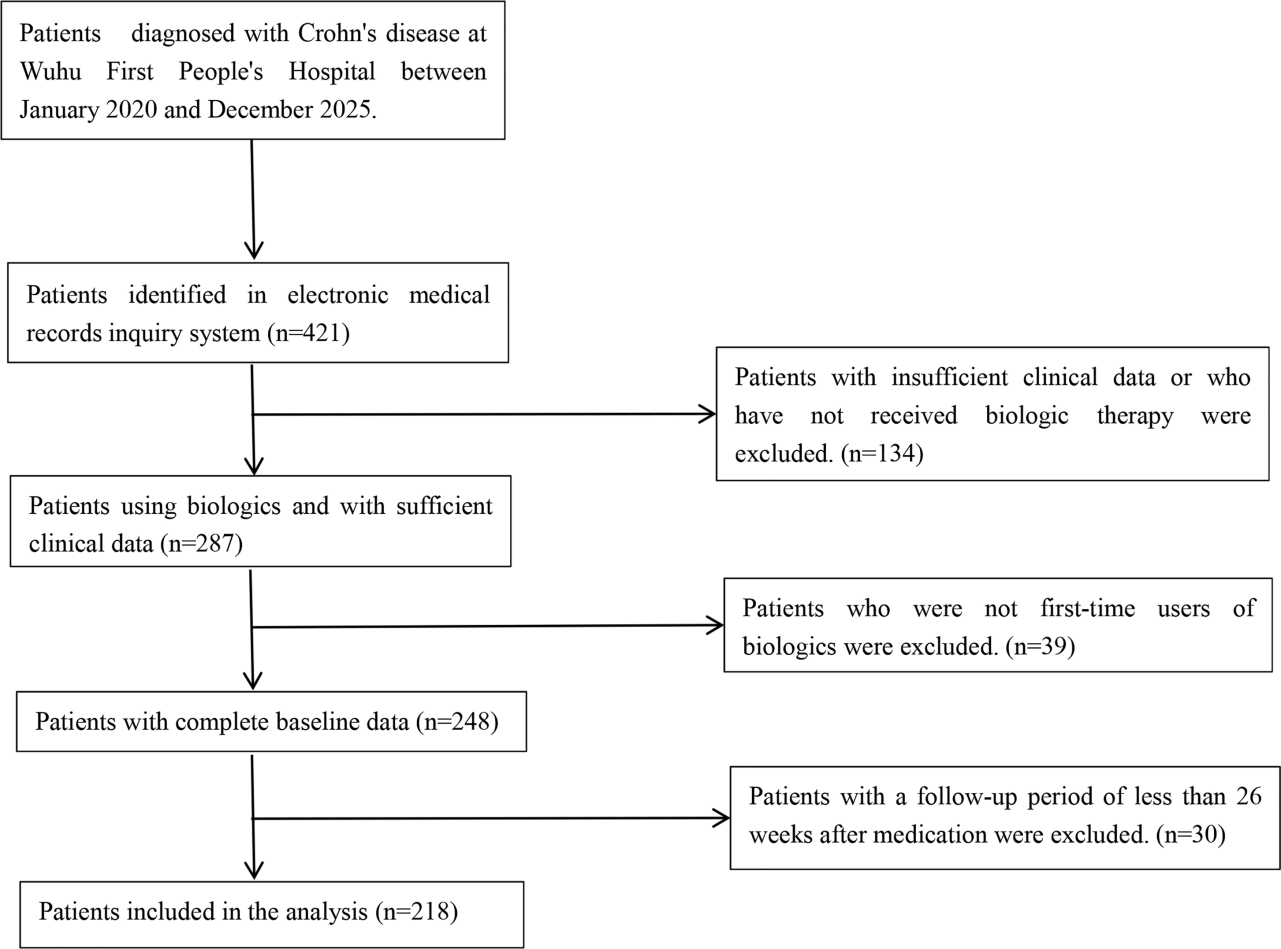
Flowchart of screening patients.

Initially, 248 individuals with CD who underwent biologic or targeted therapy were collected. 30 individuals who did not reach the study endpoint were excluded. Eventually, 218 individuals were enrolled in the analysis. The flowchart of screening participants during the study period is shown in **Figure 1**. They were grouped according to different treatment regimens into the IFX treatment group (n=97), UST treatment group (n=81), VDZ treatment group (n=23), and UPA treatment group (n=17). A total of 19 baseline variables were statistically analyzed in this study. There were statistically significant differences among the four groups in age at diagnosis, lesion site, CRP, ALB, ESR, PLT, and previous use of glucocorticoids. On the contrary, no statistically significant differences were noted in sex, BMI, pre-treatment CDAI, pre-treatment HBI, course of disease, history of oxygen therapy, disease behavior, perianal lesions, Hb, LYM, PLR, and PCT (P>0.05). Baseline demographic characteristics are depicted in **Table 1**.

**Table 1:** Comparison of demographic and clinical characteristics of patients in different treatment groups.

Characteristic	Level	IFX (n=97)	UPA (n=17)	UST (n=81)	VDZ (n=23)	P-value
Sex(n,%)						0.469
	Male	67 (69.1)	12 (70.6)	54 (66.7)	12 (52.2)	
	Female	30 (30.9)	5 (29.4)	27 (33.3)	11 (47.8)	
BMI,median(IQR)(kg/m^2^)		19.50 [18.10, 20.80]	20.70 [19.10, 25.40]	20.30 [18.50, 22.60]	19.60 [18.75, 22.20]	0.078
Before CDAI,median(IQR)		169.90 [159.80, 189.50]	174.30 [161.60, 197.30]	175.40 [165.10, 192.60]	166.70 [160.95, 179.05]	0.142
Before HBI,median(IQR)		5.00 [5.00, 6.00]	5.00 [5.00, 6.00]	6.00 [5.00, 6.00]	5.00 [5.00, 6.00]	0.363
Course,median(IQR)		4.50 [3.80, 7.00]	5.40 [3.40, 7.00]	5.00 [3.00, 7.00]	4.40 [3.95, 8.40]	0.898
Smoking(n,%)						0.221
	No	79 (81.4)	10 (58.8)	64 (79.0)	18 (78.3)	
	Yes	18 (18.6)	7 (41.2)	17 (21.0)	5 (21.7)	
Age(n,%)						0.033
	A1	10 (10.3)	0 (0.0)	1 (1.2)	0 (0.0)	
	A2	69 (71.1)	14 (82.4)	66 (81.5)	15 (65.2)	
	A3	18 (18.6)	3 (17.6)	14 (17.3)	8 (34.8)	
Site of lesion(n,%)						0.022
	L1	35 (36.1)	1 (5.9)	30 (37.0)	9 (39.1)	
	L2	6 (6.2)	3 (17.6)	1 (1.2)	3 (13.0)	
	L3	56 (57.7)	13 (76.5)	50 (61.7)	11 (47.8)	
Disease behavior(n,%)						0.888
	B1	54 (55.7)	9 (52.9)	49 (60.5)	15 (65.2)	
	B2	23 (23.7)	4 (23.5)	18 (22.2)	6 (26.1)	
	B3	20 (20.6)	4 (23.5)	14 (17.3)	2 (8.7)	
Perianal lesions(n,%)						0.911
	No	38 (39.2)	7 (41.2)	36 (44.4)	10 (43.5)	
	Yes	59 (60.8)	10 (58.8)	45 (55.6)	13 (56.5)	
CRP,median(IQR)(mg/L)		16.45 [11.60, 31.60]	8.00 [6.50, 31.18]	8.21 [2.50, 16.80]	10.21 [2.50, 18.84]	<0.001
Hb,median(IQR)(g/L)		125.00 [110.00, 138.00]	129.00 [115.00, 143.00]	131.00 [120.00, 147.00]	132.00 [120.50, 137.50]	0.153
ALB,median(IQR)(g/L)		39.70 [37.50, 41.70]	40.80 [38.20, 45.40]	41.30 [39.00, 45.20]	40.80 [38.55, 44.65]	0.015
ESR,median(IQR)(mm/L)		26.00 [21.00, 33.00]	22.00 [10.00, 34.00]	18.00 [9.00, 29.00]	18.00 [8.50, 30.50]	0.004
PLT,median(IQR) (×109/L)		317.00 [281.00, 355.00]	324.00 [257.00, 329.00]	269.00 [217.00, 330.00]	291.00 [225.50, 311.00]	0.001
LYM,median(IQR)		1.32 [1.12, 1.78]	1.50 [1.23, 1.81]	1.23 [0.97, 1.66]	1.32 [1.06, 1.53]	0.094
PLR,median(IQR)		226.35 [171.68, 283.33]	193.58 [162.40, 208.94]	210.27 [157.14, 294.12]	200.00 [154.55, 252.18]	0.16
PCT,median(IQR)		0.27 [0.22, 0.32]	0.28 [0.25, 0.30]	0.26 [0.22, 0.30]	0.24 [0.22, 0.27]	0.265
GC(n,%)						0.005
	No	68 (70.1)	6 (35.3)	62 (76.5)	13 (56.5)	
	Yes	29 (29.9)	11 (64.7)	19 (23.5)	10 (43.5)	

BMI, body mass index; Before CDAI, Crohn’s disease activity index before treatment; Before HBI, The Harved-Bradshaw index before treatment; Course, course of disease; CRP, C-reactive protein; Hb, hemoglobin; ESR, erythrocyte sedimentation rate; PLT, platelet; LYM, lymphocyte count; PLR, platelets to lymphocytes ratio; PCT, plateletcrit; GC, glucocorticoid.

### 3.2 Clinical remission rates of different biologics

**Figure 2:**
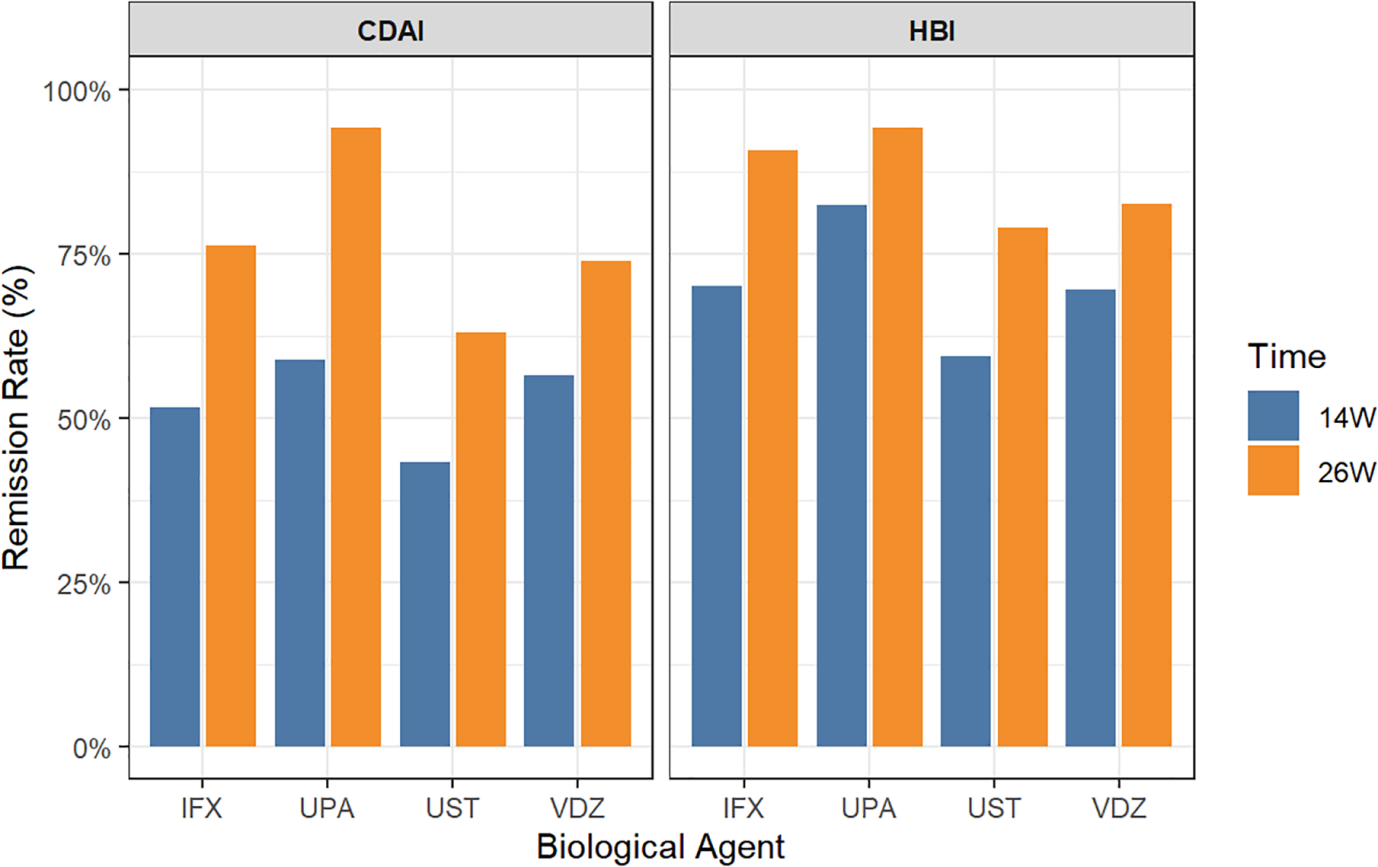
Clinical remission rates of different treatment regimens.

Statistical analysis was performed on the clinical remission rates of the four groups. Four outcome variables were analyzed: CDAI at week 14, CDAI at week 26, HBI at week 14, and HBI at week 26. Among them, a significant difference in the CDAI score at week 26 was observed among treatment groups (P = 0.023), and UPA showed the highest clinical remission rate at 94.1% (**Table 2**; **Figure 2**). Pairwise chi-square test was conducted to further ascertain the difference in efficacy between various treatment groups. The results indicated a statistically significant difference between UPA and UST (P = 0.012), while no significant differences were noted among the other groups (P>0.05) (**Table 3**).

**Table 2:** Clinical remission rate of different treatment regimens.

Outcome	IFX (n=97)	UPA (n=17)	UST (n=81)	VDZ (n=23)	P-value
CDAI (14W)					0.477
Yes	50 (51.5%)	10 (58.8%)	35 (43.2%)	13 (56.5%)	
No	47 (48.5%)	7 (41.2%)	46 (56.8%)	10 (43.5%)	
CDAI (26W)					0.023
Yes	74 (76.3%)	16 (94.1%)	51 (63%)	17 (73.9%)	
No	23 (23.7%)	1 (5.9%)	30 (37%)	6 (26.1%)	
HBI (14W)					0.2
Yes	68 (70.1%)	14 (82.4%)	48 (59.3%)	16 (69.6%)	
No	29 (29.9%)	3 (17.6%)	33 (40.7%)	7 (30.4%)	
HBI (26W)					0.099
Yes	88 (90.7%)	16 (94.1%)	64 (79%)	19 (82.6%)	
No	9 (9.3%)	1 (5.9%)	17 (21%)	4 (17.4%)	

**Table 3:** Pairwise comparison of efficacy among treatment groups.

Class	P-value
IFX vs UPA	0.096
IFX vs UST	0.053
IFX vs VDZ	0.811
UPA vs UST	0.012
UPA vs VDZ	0.096
UST vs VDZ	0.33

### 3.3 Changes in disease activity before and after treatment

The changes in disease activity before and after treatment in different treatment regimens were analyzed. There were statistical differences in CDAI scores and HBI scores before and after treatment in each group (P < 0.05). Among them, the mean ± standard deviation of CDAI before the treatment of UPA was 184.759 ± 29.578. The mean ± standard deviation of CDAI at week 26 was 132.829 ± 13.110 (**Table 4**).

**Table 4:** Changes in scores before and after treatment in each treatment group.

Outcome	IFX	UPA	UST	VDZ	P-value
CDAI(14W)					<0.05
Prior treatment	178.403 ± 25.528	184.759 ± 29.578	186.086 ± 32.026	171.813 ± 14.991	
Posttreatment	152.038 ± 23.888	149.382 ± 12.162	161.032 ± 28.952	142.800 ± 22.126	
CDAI(26W)					<0.05
Prior treatment	178.403 ± 25.528	184.759 ± 29.578	186.086 ± 32.026	171.813 ± 14.991	
Posttreatment	142.121 ± 27.756	132.829 ± 13.110	152.910 ± 25.764	134.139 ± 23.131	
HBI(14W)					<0.05
Prior treatment	5.65 ± 1.217	5.71 ± 1.263	5.89 ± 1.255	5.48 ± 0.790	
Posttreatment	4.06 ± 0.922	3.88 ± 0.928	4.21 ± 0.904	4.04 ± 0.767	
HBI(26W)					<0.05
Prior treatment	5.65 ± 1.217	5.71 ± 1.263	5.89 ± 1.255	5.48 ± 0.790	
Posttreatment	3.22 ± 1.002	2.94 ± 1.088	3.58 ± 0.986	3.17 ± 1.029	

### 3.4 Selection of predictive variables

**Figure 3:**
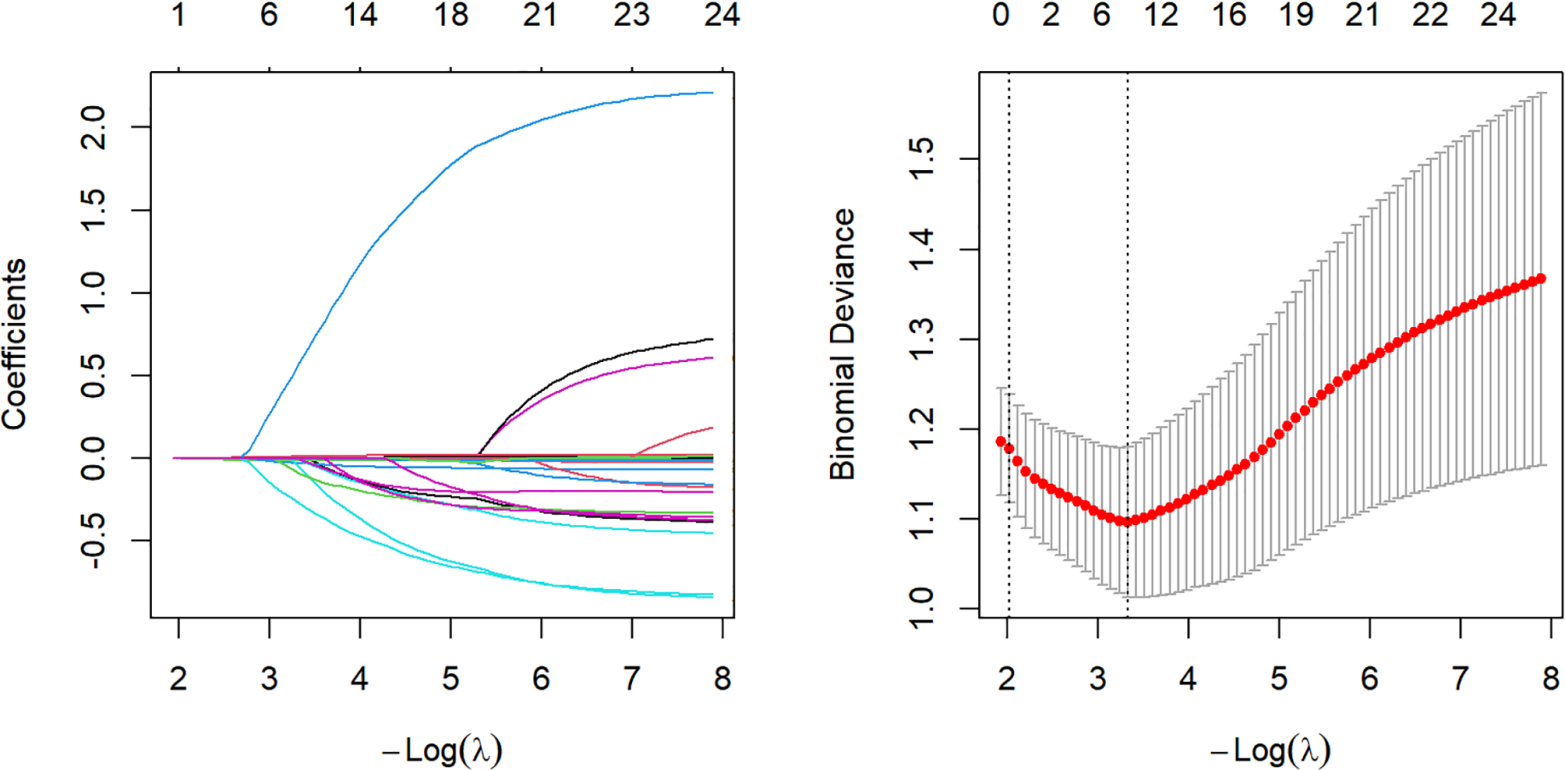
Variable Selection. LASSO regression analysis was used, with 10-fold cross-validation. A coefficient profile plot was drawn based on the logarithmic (Lambda) sequence (left panel). The tuning parameter (Lambda) for LASSO regression bias was selected based on the minimum absolute contraction and selection operator (left dashed line) and the 1-SE standard (right dashed line) (right panel). LASSO, least absolute shrinkage and selection operator; SE, standard error.

In this study, the clinical remission rate of CDAI at week 26 was used as the outcome variable to select predictive variables by LASSO regression. Based on lambda.min, predictive factors were screened. Nine variables were found to be potential predictors of clinical remission, including CDAI score before treatment, course of disease, smoking history, lesion site, disease behavior, perianal lesions, Hb, ESR, and type of biologic (**Figure 3**).

### 3.5 Nomogram construction

**Figure 4:**
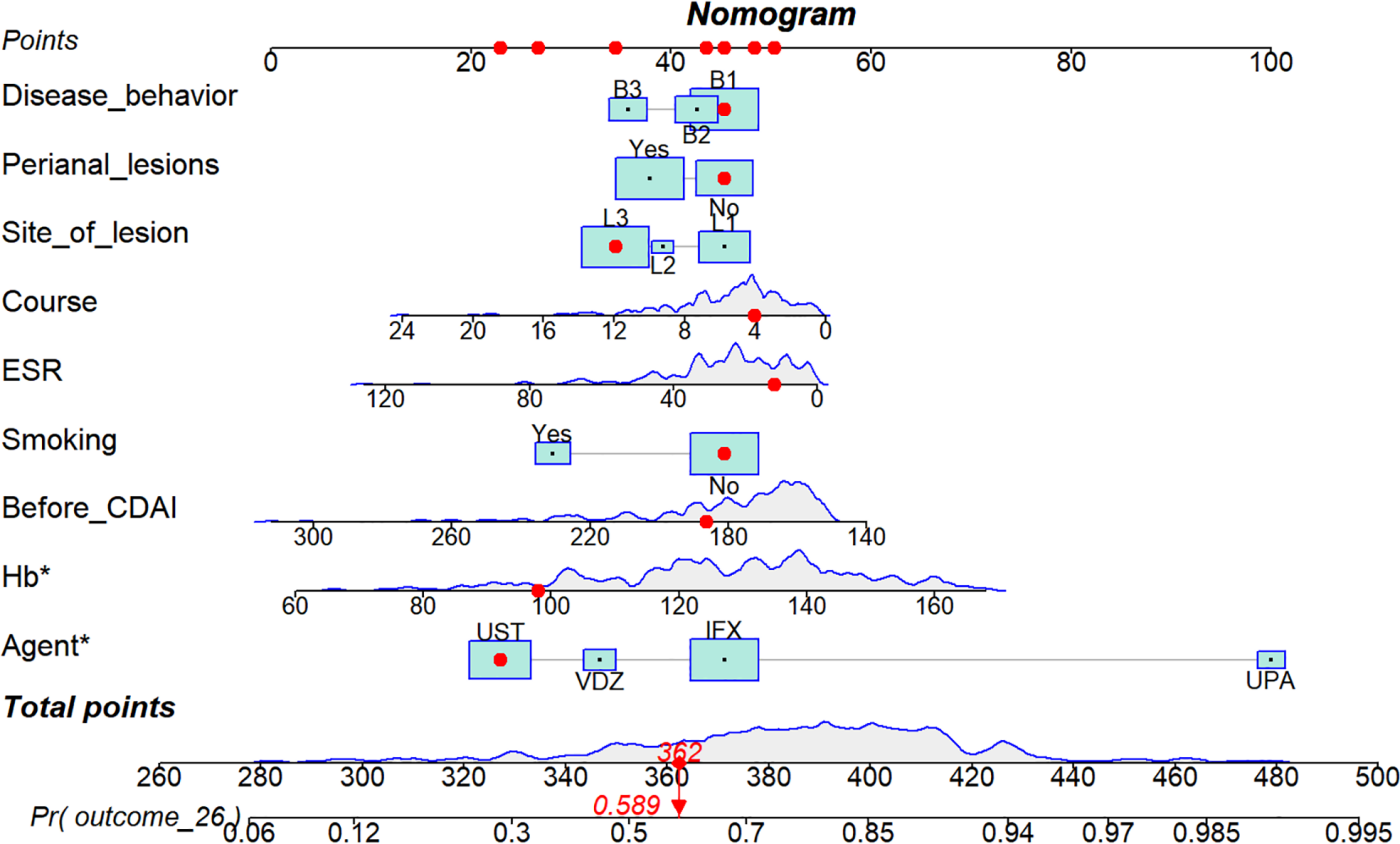
Nomogram for predicting clinical remission rates at week 26 in patients with Crohn’s disease receiving biologics or targeted therapies. According to this nomogram, if a patient has a 4-year course of disease, no perianal involvement, disease behavior of type B1, disease site of L3, no smoking history, a pre-treatment CDAI score of 186.4, erythrocyte sedimentation rate of 12 mm/H, hemoglobin of 98 g/L, and UST inhibitor therapy, the estimated probability of achieving clinical remission after induction therapy is 0.589. Green squares represent categorical variables, and the size of the square represents the proportion of variables in the overall sample. The purple curve represents the distribution of continuous variables in the overall sample. B1, non-stenotic, non-penetrating type; L3, large and small bowel type; CDAI, Crohn’s disease activity index; UST, ustekinumab.

A nomogram was constructed to visualize the prediction model. The first patient was used as an example to show how to use the nomogram. The results showed that when the patient had a disease course of 4 years, no perianal involvement, disease behavior of no stenosis and no perforation (B1), lesion site of large intestine and small intestine type (L3), no history of smoking, UST treatment, CDAI score of 186.4 before treatment, ESR of 12 mm/H, Hb of 98 g/L, the probability of achieving clinical remission after induction therapy was estimated to be 0.589 (**Figure 4**).

### 3.6 Model evaluation and validation

**Figure 5:**
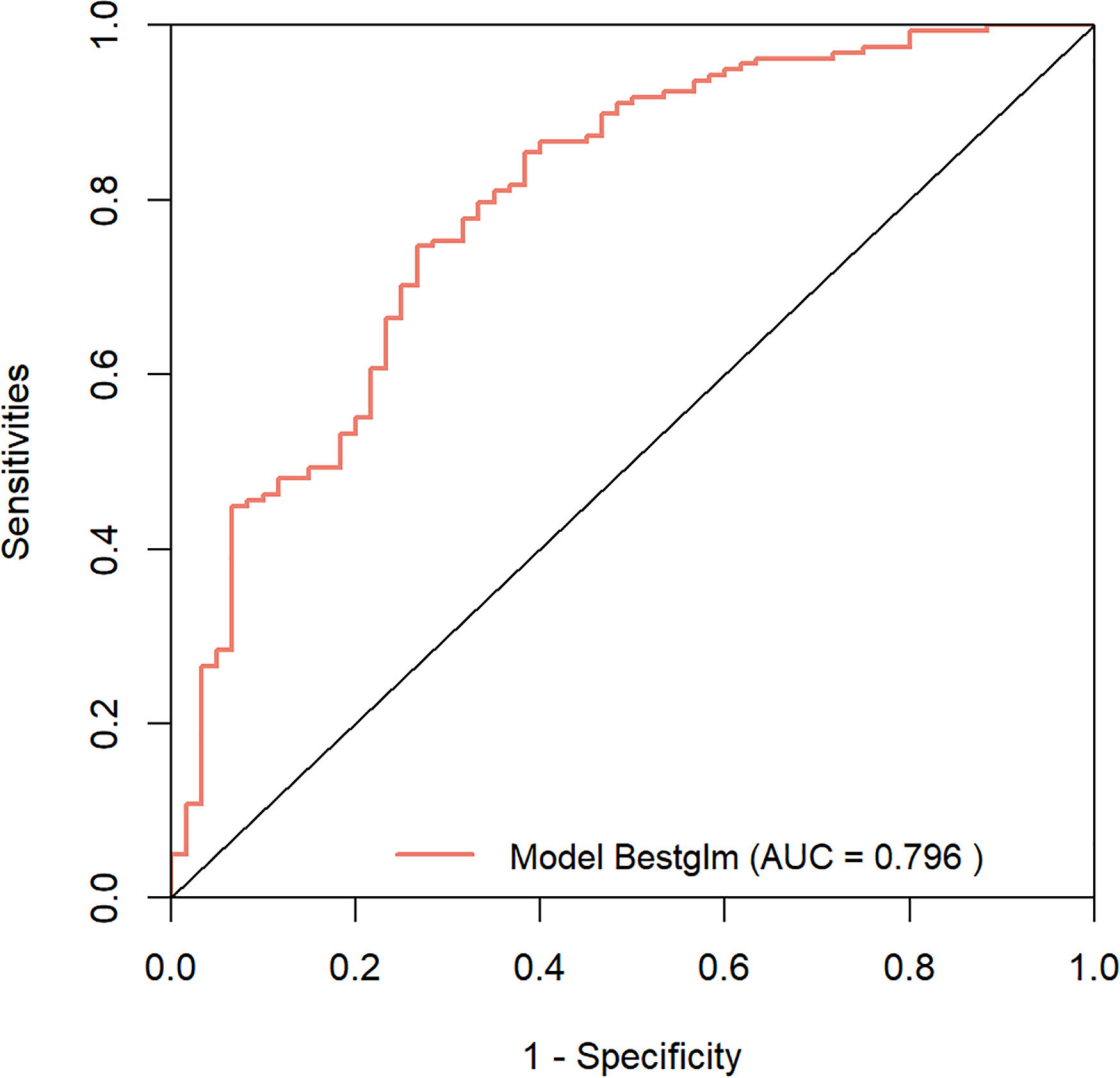
Receiver operating characteristic (ROC) curves of the prediction model. The area under the ROC curve for the model is 0.796 (95% confidence interval: 0.728-0.865), marked in orange.

**Figure 6:**
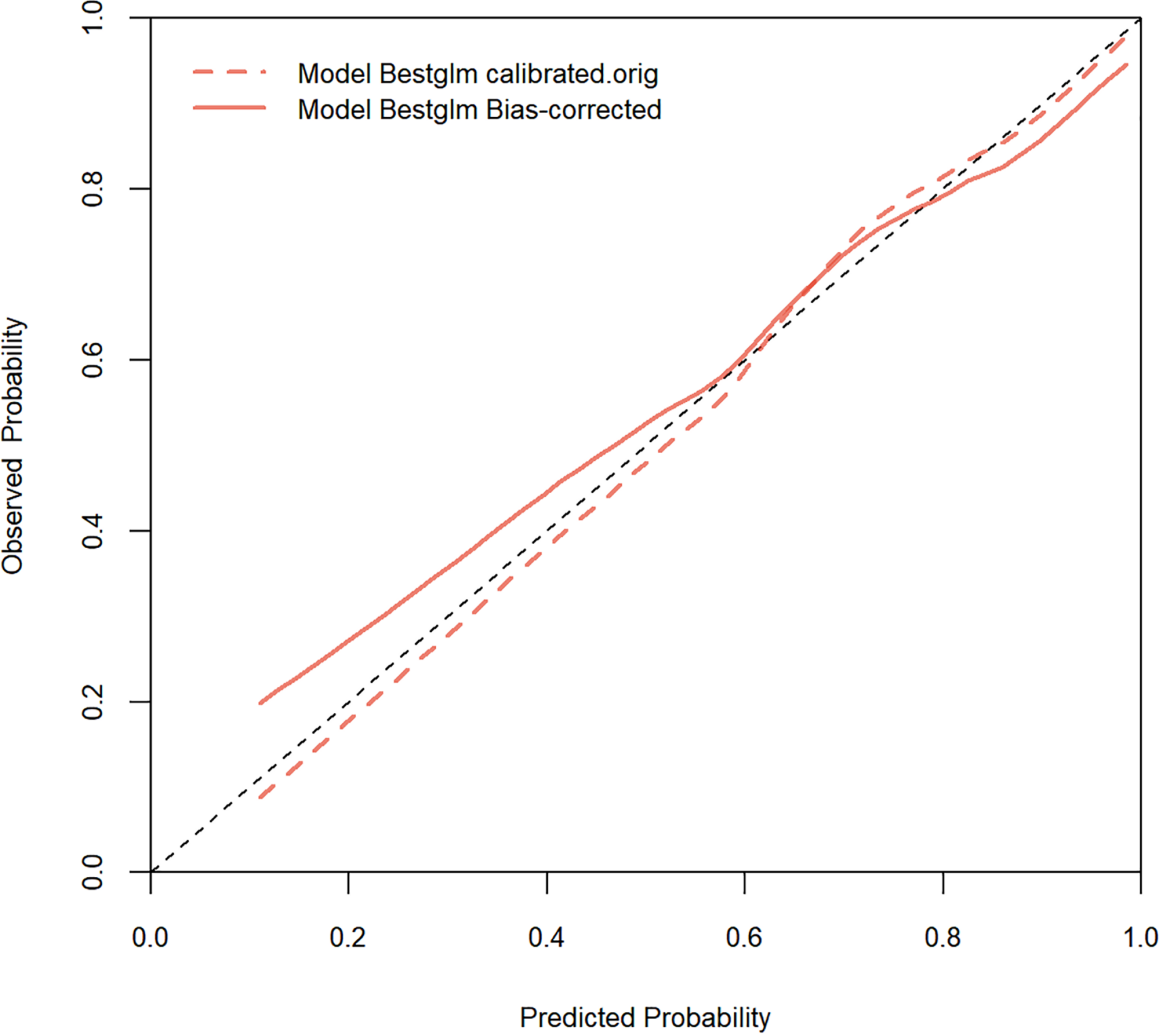
Calibration curve of the prediction model. The orange dashed line is the original calibration curve of the model (without bias correction). The orange solid line is the calibration curve of the model after bias correction. The calibration curve of the model fluctuates around the diagonal, indicating good calibration.

**Figure 7:**
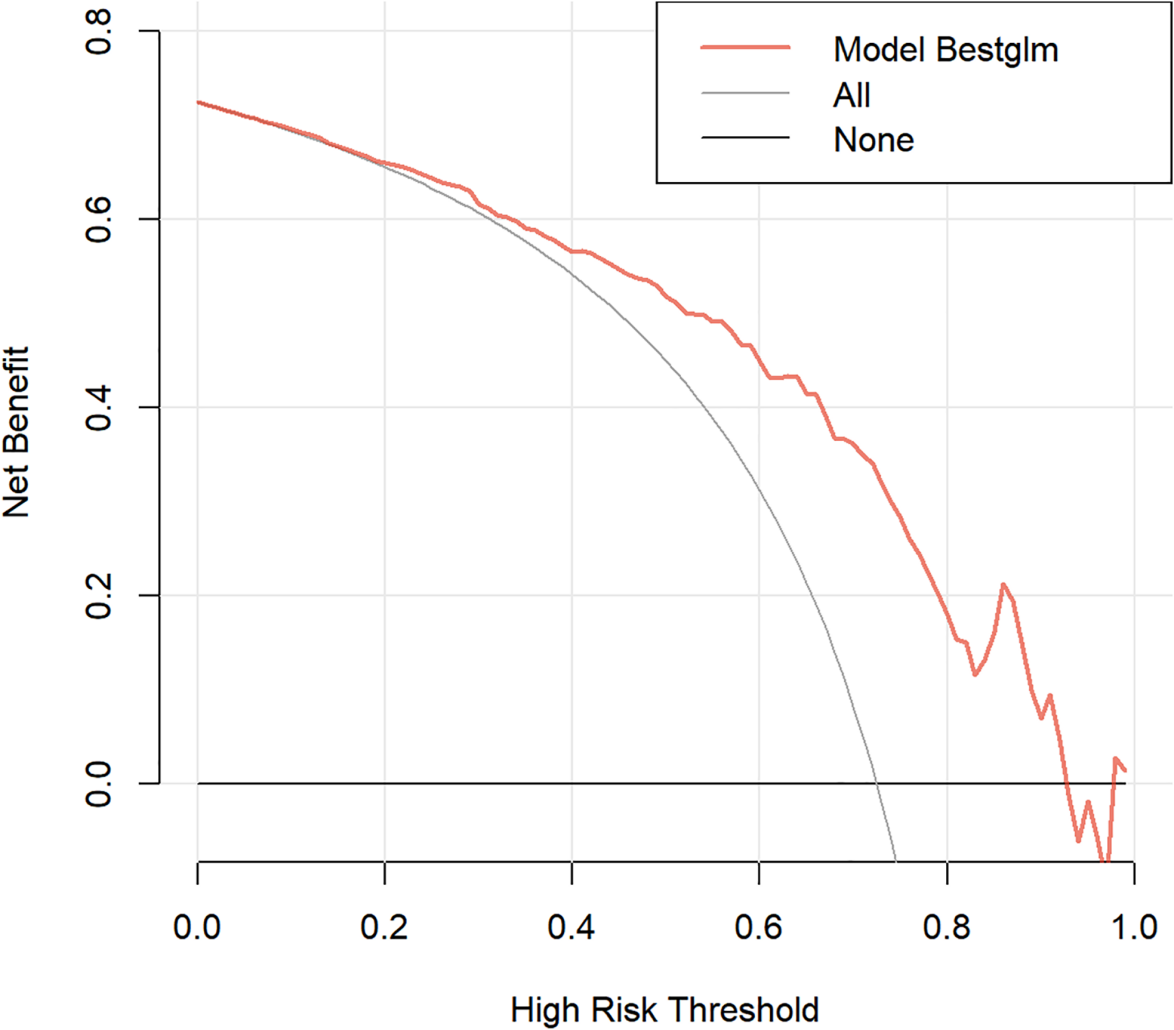
Decision curve of the prediction model for predicting clinical remission. The orange line represents the decision curve of the model. The “None” line represents the net benefit obtained without any intervention. The “All” line represents the net benefit obtained with all interventions. The decision curve demonstrates that using the model to predict clinical remission rates provides greater benefit than either the full treatment strategy or the no-treatment strategy.

We developed a prediction model based on the nine variables. The ROC of the model was 0.796 (95% confidence interval [CI]: 0.728-0.865)(**Figure 5**), indicating that the model had favorable discrimination performance. In addition, the calibration curves of the model fluctuated around the diagonal, indicating good calibration (**Figure 6**). DCA curve of the model was above the two reference lines, indicating that the clinical applicability of the model was good (**Figure 7**).

**Figure 8:**
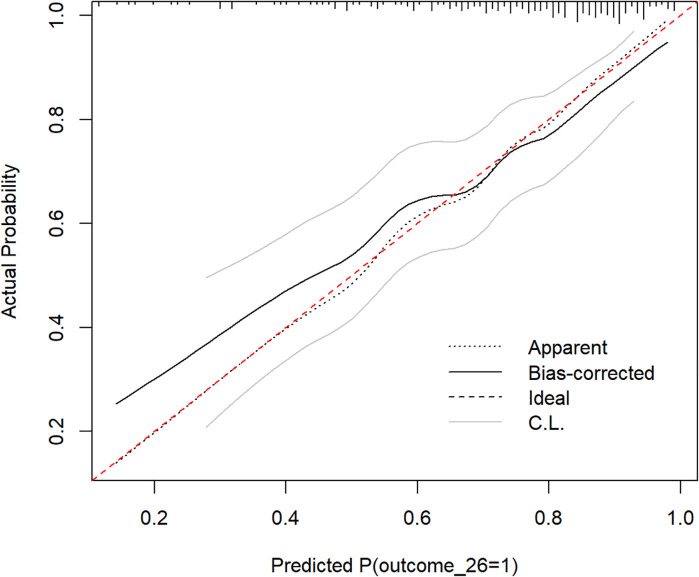
Calibration curve of the prediction model in internal validation. The calibration curve of the prediction model is obtained using the bootstrap method with 1000 repeated samples. The red dashed line represents the ideal calibration line, and the black dashed line represents the uncalibrated curve of the original model. The black solid line represents the calibration curve after bias correction. CL represents the upper and lower boundaries of the curve after bias correction.

This study used the C-index and bootstrap method to repeatedly sample over 1000 iterations to determine the generalization ability of the model in internal validation. The results suggested that the C-index was 0.74 in the internal validation set, implying that the model had moderate generalizability. The calibration curves in the internal validation set fluctuated around the diagonal, indicating that the model had good calibration (**Figure 8**).

### 3.7 Sensitivity analysis

To further verify the robustness of the results, sensitivity analyses was conducted on the glucocorticoid exposure group and the non-exposure group. The results showed that the clinical remission rates of different treatment regimens exhibited a consistent trend in both the glucocorticoid exposure and non-exposure groups (**Supplementary Tables 1**, **2**). Further multiple comparisons revealed statistically significant differences in the clinical remission rates of UPA and UST in both the non-exposure and exposure groups (**Supplementary Tables 3**, **;4**), indicating the robustness of the results.

## 4 Discussion

This study compared the clinical remission rates of different biologics and targeted therapies. The results indicated statistically significant differences in CDAI scores at week 26 among various regimens, and UPA showed the highest clinical remission rate (94.1%). Furthermore, a model was built to forecast the clinical efficacy of biologics or targeted therapy in individuals with CD. The model included nine variables, encompassing pre-treatment CDAI score, course of disease, smoking history, lesion site, disease behavior, perianal lesions, Hb, ESR, and type of biologic or targeted therapy. The AUC of the model was 0.796, and both the calibration curve and DCA curve indicated that the model had good calibration and clinical applicability, providing a reference for precision treatment of CD.

CD is a subtype of inflammatory bowel disease that may influence the entire digestive tract. Its clinical manifestations are complex and diverse. As the disease progresses, it can cause complications such as intestinal obstruction, intestinal fistula, abdominal abscess, and even cancer, severely impacting patients’ health and quality of life. CD is intractable, and lifelong treatment is required, leading to a significant financial burden on patients. In China, the average treatment cost for CD is about 54,000 RMB, and patients themselves pay 55.6% of the cost (19, 20). In recent years, biologics and small-molecule drugs have been developed and used in the treatment of CD. Although various treatment options are available in clinical practice, clinical data are still lacking to provide definitive guidance for selecting treatment regimens for CD in clinical practice.

This study investigated the clinical remission rates of different biologics and targeted therapy. UPA was found to have the highest clinical remission rate, reaching 94.1% at week 26. This result is largely consistent with the results of a multicenter study by Jalpa Devi, which shows a 97.7% clinical remission rate at 6 months in patients receiving advanced UPA treatment (21). However, several international studies (22–24) have shown that the clinical remission rate of UPA fluctuates between 48% and 86.8%, possibly due to differences in geographical location, lifestyle, regional medical conditions, and economic status. Furthermore, differences in clinical remission criteria among medical institutions may also affect remission outcomes. This study showed a clinical remission rate of 94.1% in the UPA treatment group. Given the limited number of cases included in this study, this finding needs to be verified in a larger-scale study in the future. IFX had the second-highest clinical remission rate, reaching 76.3% at week 26. This result is largely consistent with the findings of a retrospective study by Sprake S MB et al., which shows that 65.9% of patients have achieved clinical remission. At week 26, the clinical remission rate with UST was 63%. A multicenter prospective study in China has reported a 64.9% clinical remission rate at week 20 (25), which is similar to the results obtained in this study. The clinical remission rate at week 26 in the VDZ treatment group was 73.9%. This result is not significantly different from the results of a prospective study in Taiwan, which reports a 71.4% clinical remission rate in individuals with CD (26). This study preliminarily explored the effectiveness of different biologics and targeted therapies using real-world clinical data. The results showed that UPA had the highest clinical remission rate, providing clinicians with some clinical evidence to choose treatment options. These findings suggest that UPA has great potential as an emerging treatment option for CD. In this cohort, patients receiving UPA showed a high clinical remission rate (94.1%). However, due to the small sample size, this result should be considered a preliminary observation and needs to be validated in a larger sample study.

This study used LASSO regression to screen for potential predictors of response to biologic or targeted therapy. The results showed that pre-treatment CDAI score, course of disease, smoking history, lesion site, disease behavior, perianal lesions, Hb, ESR, and type of biologic or targeted therapy were associated with clinical remission after biologic or targeted therapy in CD. Perianal lesions, large and small intestinal lesions, and stenosis or penetrating lesions were found to be negative predictors of clinical remission after induction therapy. Patients with perianal involvement and large and small intestinal lesions tend to have more extensive lesions, while patients with penetrating or stenotic lesions tend to have more severe and complex conditions. Therefore, it is more difficult to treat these populations, and theoretically, their clinical remission rate after biologic or targeted therapy is also lower (27–29). Studies on the natural course of CD have shown that a longer disease course may induce more severe and complex conditions, such as intestinal perforation and enterocutaneous fistula. Therefore, there may be an association between the course of disease and disease severity, which in turn results in a decrease in clinical remission rate after treatment with biologic or targeted therapy (30). Its results are consistent with our findings, implying that the course of disease is a negative predictor of clinical remission after induction therapy.

This study suggested that higher baseline Hb levels were a positive predictor of clinical remission after biologic or targeted therapy, while higher baseline ESR levels were a negative predictor. This is consistent with the findings of several studies, both domestically and internationally (31–33). Furthermore, this study found that a high baseline CDAI score was a negative predictor of clinical remission rate, consistent with previous clinical studies both domestically and internationally (34, 35). Multiple studies have demonstrated that a higher baseline CDAI indicates a lower clinical remission rate after biologic or targeted therapy. Domestic and international studies have shown that smoking is a high-risk factor for treatment failure in CD; compared with non-smokers, smokers have a lower clinical remission rate after biologic therapy (36, 37). This is consistent with the findings of this study, suggesting that smoking is a negative predictor of clinical remission. Studies have demonstrated a negative association between higher BMI and induction therapy (38, 39), while another investigation reveals that lower baseline BMI is a negative predictor of clinical remission (40). However, this study yielded negative results, mainly because the number of cases included in the study was limited and the difference in BMI between the treatment groups was not statistically significant. Further research is needed to clarify their relationship.

In terms of the strengths of this study, the variables used in the model are readily accessible and simple, with no need for invasive methods such as endoscopy or histological examination. This helps enhance patient acceptance and lower costs. The model may be used to support clinical decision-making, and its predictive results may guide clinicians in developing individualized treatment plans, thereby improving long-term patient outcomes. This study aligns with the ECCO initiative and can promote precision medicine (41).

Our study has certain limitations. First, the sample size is limited, particularly in the UPA and VDZ treatment groups, and this is a single-center, retrospective, preliminary exploratory study on individuals with CD in China. Further validation with multiple centers and larger samples from surrounding areas is needed. Second, clinical remission is not the optimal treatment endpoint recommended by guidelines. Mucosal healing and transmural healing should be considered as deeper treatment goals, and endoscopic and histological data should be included in future studies. Nonetheless, we believe that clinical remission should be the initial goal of clinical treatment. Furthermore, there are unmeasured confounding factors, and the model performance was moderate (C-index of 0.74). Eventually, this study only conducted internal validation through repeated sampling. No external validation is performed to assess the accuracy and reliability of the constructed model, which limits its generalizability. Furthermore, this study primarily focuses on short-term efficacy (within 14 weeks and 26 weeks). Further follow-up observation is needed to explore the long-term efficacy and safety of biologics and targeted therapy.

## 5 Conclusion

This study reveals that UPA has the highest clinical remission rate based on CDAI scores at week 26. Furthermore, a simple and reliable predictive tool has been developed for predicting response to biologics and targeted therapy in CD patients. The study suggests that pre-treatment CDAI score, course of disease, smoking history, lesion site, disease behavior, perianal lesions, Hb, ESR, and type of biologic or targeted therapy are predictors of clinical remission. The AUC of the constructed model is 0.796. The predictive model will help clinicians identify patients expected to respond well to biologics and targeted therapy, assist in developing individualized treatment plans, and promote precision medicine. In addition, by forecasting patient treatment response before medication, identifying patients with low response rates and adjusting treatment strategies accordingly can avoid treatment delays and improve long-term patient outcomes.

## Data Availability

The raw data supporting the conclusions of this article will be made available by the authors, without undue reservation.

## Supplementary material

Supplementary Table 1 Clinical Remission Rates in Different Treatment Regimens Among Non-Exposed Groups for Glucocorticoids.

Supplementary Table 2 Clinical Remission Rates for Different Treatment Regimens in the Glucocorticoid Exposure Cohort.

Supplementary Table 3 Pairwise comparisons of treatment efficacy between non-exposed groups of glucocorticoids.

Supplementary Table 4 Pairwise comparisons of treatment efficacy among glucocorticoid-exposed groups.
