# Supplementary material for: Development and validation of a predictive nomogram for response to biologics and targeted therapy in Crohn’s disease: a retrospective cohort study
Source: Front Immunol. 2026 Apr 16;17:1788290. doi: 10.3389/fimmu.2026.1788290 (PMC13128662; doi:10.3389/fimmu.2026.1788290)
Supplement: Supplementary Table 1 — Clinical Remission Rates in Different Treatment Regimens Among Non-Exposed Groups for Glucocorticoids. [file Table1.docx]

**Supplementary materials**

**Table S1** Clinical Remission Rates in Different Treatment Regimens Among Non-Exposed Groups for Glucocorticoids

|  | IFX(68) | UPA(6) | UST(62) | VDZ(13) | P |
| --- | --- | --- | --- | --- | --- |
| CDAI(14W) |  |  |  |  | 0.218 |
| Yes | 36(52.9%) | 4(66.7%) | 26(41.9%) | 9(69.2%) |  |
| No | 32(47.1%) | 2(33.3%) | 36(58.1%) | 4(30.8%) |  |
| CDAI(26W) |  |  |  |  | 0.046 |
| Yes | 53（77.9%） | 6（100%） | 42（67.7%） | 12（92.3%） |  |
| No | 15（22.1%） | 0（0.0%） | 20（32.3%） | 1（7.7%） |  |
| HBI(14W) |  |  |  |  | 0.2 |
| Yes | 51（75.0%） | 5（83.3%） | 37（59.7%） | 10（76.9%） |  |
| No | 17（25.0%） | 1（16.7%） | 25（40.3%） | 3（23.1%） |  |
| HBI(26W) |  |  |  |  | 0.058 |
| Yes | 64（94.1%） | 6（100%） | 51（82.3%） | 13（100%） |  |
| No | 4（5.9%） | 0（0.0%） | 11（17.7%） | 0（0.0%） |  |

**Table S2** Clinical Remission Rates for Different Treatment Regimens in the Glucocorticoid Exposure Cohort

|  | IFX(29) | UPA(11) | UST(62) | VDZ(13) | P |
| --- | --- | --- | --- | --- | --- |
| CDAI(14W) |  |  |  |  | 0.93 |
| Yes | 14(48.3%) | 6(54.5%) | 9(47.4%) | 4(40.0%) |  |
| No | 15(51.7%) | 5(45.5%) | 10(52.6%) | 6(60.0%) |  |
| CDAI(26W) |  |  |  |  | 0.043 |
| Yes | 21（72.4%） | 10（90.9%） | 9(47.4%) | 5（50.0%） |  |
| No | 8（27.6%） | 1（9.1%） | 10(52.6%) | 5（50.0%） |  |
| HBI(14W) |  |  |  |  | 0.507 |
| Yes | 17（58.6%） | 9（81.8%） | 11（57.9%） | 6（60.0%） |  |
| No | 12（41.4%） | 2（18.2%） | 8（42.1%） | 4（40.0%） |  |
| HBI(26W) |  |  |  |  | 0.24 |
| Yes | 24（82.8%） | 10（90.9%） | 13（68.4%） | 6（60.0%） |  |
| No | 5（17.2%） | 1（9.1%） | 6（31.6%） | 4（40.0%） |  |

**Table S3** Pairwise comparisons of treatment efficacy between non-exposed groups of glucocorticoids

| Class | P-value |
| --- | --- |
| IFX vs UPA | 0.091 |
| IFX vs UST | 0.19 |
| IFX vs VDZ | 0.233 |
| UPA vs UST | 0.036 |
| UPA vs VDZ | 0.376 |
| UST vs VDZ | 0.073 |

**Table S4** Pairwise comparisons of treatment efficacy among glucocorticoid-exposed groups

| Class | P-value |
| --- | --- |
| IFX vs UPA | 0.211 |
| IFX vs UST | 0.08 |
| IFX vs VDZ | 0.195 |
| UPA vs UST | 0.017 |
| UPA vs VDZ | 0.058 |
| UST vs VDZ | 0.893 |
